# A novel α-galactosidase from the thermophilic probiotic *Bacillus coagulans* with remarkable protease-resistance and high hydrolytic activity

**DOI:** 10.1371/journal.pone.0197067

**Published:** 2018-05-08

**Authors:** Ruili Zhao, Rui Zhao, Yishuai Tu, Xiaoming Zhang, Liping Deng, Xiangdong Chen

**Affiliations:** 1 State Key Laboratory of Virology, College of Life Sciences, Wuhan University, Wuhan, P. R. China; 2 China Center for Type Culture Collection, Wuhan, P. R. China; Weizmann Institute of Science, ISRAEL

## Abstract

A novel α-galactosidase of glycoside hydrolase family 36 was cloned from *Bacillus coagulans*, overexpressed in *Escherichia coli*, and characterized. The purified enzyme Aga-BC7050 was 85 kDa according to SDS-PAGE and 168 kDa according to gel filtration, indicating that its native structure is a dimer. With *p*-nitrophenyl-α-d- galactopyranoside (pNPGal) as the substrate, optimal temperature and pH were 55 °C and 6.0, respectively. At 60 °C for 30 min, it retained > 50% of its activity. It was stable at pH 5.0–10.0, and showed remarkable resistance to proteinase K, subtilisin A, α-chymotrypsin, and trypsin. Its activity was not inhibited by glucose, sucrose, xylose, or fructose, but was slightly inhibited at galactose concentrations up to 100 mM. Aga-BC7050 was highly active toward pNPGal, melibiose, raffinose, and stachyose. It completely hydrolyzed melibiose, raffinose, and stachyose in < 30 min. These characteristics suggest that Aga-BC7050 could be used in feed and food industries and sugar processing.

## Introduction

As important oil seed crops, soybeans are an abundant source of protein. In addition to being rich in protein and plant oil, soybeans also exert various health benefits, helping to reduce the incidence of human diseases such as heart-disease and cancer. Therefore, soybeans are widely used in human food and animal feed. Nevertheless, this food item contains a high content of α-galactosides, mainly including raffinose and stachyose, which monogastric animals and humans are unable to digest [1]. These compounds are digested by anaerobic bacteria in the large intestine, ultimately resulting in the production of carbon dioxide, hydrogen and a small amount of methane gas. In monogastric animals, this causes abdominal distension, diarrhea, and indigestion, along with a decrease in feed intake and growth performance [2].

α-Galactosidase (EC 3.2.1.22), also called melibiase, is used to catalyze the removal of terminal nonreducing galactose residues from different substrates. These enzymes have been widely found in microorganisms, mammals, and plants [3–5]. According to the sequence similarity, they can be classified into glycoside hydrolase (GH) 4, 27, 36, 57, 97, and 110 families in the CAZy database. The hydrolysis ability of α-galactosidases makes them desirable for various biotechnology applications, particularly in the feed and sugar industries. Since the raffinose family oligosaccharides (RFOs) from soybean meal cause flatulence and gastrointestinal discomfort, α-galactosidases are necessary to digest these sugars and, thus, serve to moderate flatulence and increase the nutritional value of animal feed [6]. α-Galactosidases also have utility in sugar production, where they can hydrolyze the raffinose from beet sugar syrups, increasing the output of crystallized sugar [7]. Fabry disease, an inherited lysosomal storage disease of glycosphingolipid metabolism caused by deficient or absent α-galactosidase A activity [8], is mitigated by the introduction of α-galactosidases [9]. α-Galactosidases can also be applied to convert blood from group B to group O by removing the terminal galactose residues from glycans on the surface of B-type red blood cells [10].

To improve the nutritional value of products and make them easily digestible, α-galactosidases are often supplemented, together with proteases, to eliminate oligosaccharides (mainly raffinose and stachyose) from food, feed [2]. These enzymes must resist hydrolysis by various gut proteases, which are abundant, to properly function in the animal gut. Furthermore, the host cells used to express these proteins often produce different intracellular and extracellular proteases during industrial processes. To guarantee high output, the hydrolysis of α-galactosidases by proteases should be avoided. Therefore, highly efficient α-galactosidases with protease resistance are urgently needed for feed processing, and the food industry, among other applications. However, only a few α-galactosidases have been identified with this characteristic, and most are isolated from fungi [11–16], with relatively few originating from bacteria [17–20].

*Bacillus* could be considered an ideal candidate source of protease-resistant resources. This genus itself can produce multiple proteases such as subtilisin A, alkaline protease, neutral protease, and keratinase, among others. Some of these function in the intracellular space, whereas others function on the cell membrane or extracellularly. The growth environment of *Bacillus* is complex and diverse, with a considerable portion of species existing in soil, animal intestines, and plant tissue, wherein diverse proteases can be found. Various proteins from *Bacillus* have been reported to be protease-resistant [20–25]. At present, only one protease-resistant α-galactosidase (*Bacillus megaterium*) has been reported in *Bacillu*s [20], highlighting the urgent need to develop vast resources.

We selected seven different species of *Bacillus* predicted to harbor α-galactosidase-encoding genes, including *B*. *coagulans*, *B*. *circulans*, *B*. *niacin*, *B*. *korlensis*, *B*. *kribbensis*, *B*. *muralis*, and *B*. *megaterium*, and investigated their α-galactosidase activity and resistance to protease at original host expression levels. We found that only the α-galactosidase produced by *B*. *coagulans* could strongly resist hydrolysis by protease. Therefore, we cloned and overexpressed the *B*. *coagulans* α-galactosidase gene in *Escherichia coli* for further study. This is the first report of an α-galactosidase from *B*. *coagulans*.

## Materials and methods

### Strains, vectors, and reagents

*E*. *coli* DH5α was used for propagation of plasmids and *E*. *coli* BL21 (DE3) was for expression of the α-galactosidase gene. The pET28a(+) (Merck, Darmstadt, Germany) vector was used for gene cloning. Fastdigest restriction endonucleases and T4 DNA ligase were obtained from Thermo Fisher Scientific (Waltham, MA, USA). Plasmid extraction kits and Bacteria Genomic DNA kits were procured from Tiangen (Beijing, China). Ni^2+^-NTA agarose was purchased from Qiagen (Hilden, Germany) to collect His-tagged protein. Proteinase K and trypsin were from Amresco (Solon, OH, USA), and subtilisin A and α-chymotrypsin were provided by Sigma (St. Louis, CA, USA). The centrifugal filter used to concentrate protein and the silica gel plates used for thin-layer chromatography (TLC) analysis were purchased from Merck (Darmstadt, Germany). Unless otherwise stated, all other chemicals and reagents used in this paper were of analytical grade.

### Gene cloning and heterologous expression

*B*. *coagulans* ATCC 7050 has been maintained in the China Center for Type Culture Collection (CCTCC, http://www.cctcc.org/) under the NO. CCTCC AB 92066. The α-galactosidase gene sequence of *B*. *coagulans* ATCC 7050 was downloaded from NCBI (https://www.ncbi.nlm.nih.gov/), and the locus tag is BF29_RS15105. Multiple sequence alignment and analysis were carried out using Vector NTI 11.5.1.

For extraction of the genomic DNA, *B*. *coagulans* was cultivated at 55 °C for 12 h in nutrient broth medium (10 g/L tryptone, 5 g/L yeast extract, 5 g/L NaCl). Genomic DNA was extracted from the culture using the Bacteria Genomic DNA kit. The gene encoding α-galactosidase was amplified by PCR from the genome of *B*. *coagulans* with primers Aga7050 S: 5´-TTCGCTAGCATGATTACATTTGATG-3´ and Aga7050 A: 5´-CCGCTCGAGTTACTCGTACACCGCC-3´. Restriction sites for NheI and XhoI (underlined bases) were added to the forward and reverse PCR primers respectively. The PCR amplification parameters were as follows: preincubation at 95 °C for 10 min, followed by 32 cycles of denaturation at 94 °C for 30 s, annealing at 68 °C for 30 s, and extension at 72 °C for 5 min, and then a final extension of 10 min at 72 °C. The purified PCR product was inserted into the pET28a(+) vector using the NheI and XhoI sites and T4 ligase. The ligated product was transformed into *E*. *coli* DH5α competent cells. Positive transformants containing the recombinant plasmid pET28a-AgaBC7050 were confirmed by PCR analysis and DNA sequencing. For protein expression, the propagated plasmids were extracted for introduction into the expression host *E*. *coli* BL21.

### Purification and identification of recombinant enzyme

A single colony of *E*. *coli* BL21 containing pET28a-AgaBC7050 was picked and inoculated into 5 mL nutrient broth plus 50 μg/mL kanamycin. After incubation for 12 h (200 rpm, 37 °C), 500 μL of culture was transferred into 50 mL of medium in a 250-mL flask, and incubated until the absorbance value (OD_600_) reached approximately 0.6–0.8. Then, 1 mM IPTG was added and the culture was incubated further for 6 h at 37 °C to induce protein expression. *E*. *coli* culture (50 mL) was centrifuged at 13,000 × *g* for 10 min to collect the cells, which were then disrupted by sonication. The crude cell lysates were clarified by centrifugation and filtration, and the clear filtrate was loaded onto a Ni^2+^-NTA agarose column. α-Galactosidase was eluted into washing buffer (50 mM NaH_2_PO_4_, pH 8.0, 500 mM NaCl, 200 mM imidazole), and concentrated and buffer-exchanged using McIlvaine buffer (sodium phosphate/citric acid, pH 6.0) with a 50 kDa MW cut-off centrifugal filter. The concentrated protein was preserved with 10% glycerol at -80 °C until use.

The molecular weight of Aga-BC7050 was estimated by sodium dodecyl sulfate-polyacrylamide gel electrophoresis (SDS-PAGE), which was performed with 12% separating gels and staining using Coomassie Brilliant Blue R-250. We used the Bradford method to measure protein concentrations with BSA as the standard [26]. The molecular mass of the native protein was determined using a Superdex 200 Increase 10/300 GL column (10 mm × 30 mm). Thereafter, 10 mM phosphate buffer (0.14 M NaCl, pH 7.4) was used to elute the protein, at a flow rate of 0.75 mL/min at room temperature. As molecular weight standards for gel filtration, the following proteins were used: albumin (66.0 kDa), lactate dehydrogenase (140.0 kDa), catalase (232.0 kDa), ferritin (440.0 kDa), and thyroglobulin (669.0 kDa).

### Enzyme activity assay

To determine the activity of Aga-BC7050, 100 μL of enzyme (appropriately diluted) was incubated with the substrate (5 mM pNPGal) in 200 μL of McIlvaine buffer at pH 6.0 and 55 °C for 30 min [26]. To terminate the reaction, 800 μL of 0.2 M Na_2_CO_3_ was added, and the absorbance of the product, p-nitrophenol (pNP), was measured at 400 nm [27]. One unit of enzyme activity was equal to the amount of enzyme required to produce 1 μmol of pNP per minute under the same assay conditions described previously. All enzyme activity assays were performed in triplicate and experiments were repeated at least three different times.

### Temperature and pH profiles

To determine the optimal temperature for the enzyme, α-galactosidase activity was examined in McIlvaine buffer (pH 6.0) for 30 min at temperatures ranging from 40 to 70 °C. The thermostability of Aga-BC7050 was assayed after pre-incubation without substrate at 37, 55, or 60 °C for different periods of time, and aliquots of the reaction mixture were withdrawn at time intervals to determine the residual enzyme activity under standard conditions (pH 6.0, 55 °C, 30 min).

The optimum pH of Aga-BC7050 was measured at 55 °C using McIlvaine buffer (pH range 4.0–8.0). To test pH stability, α-galactosidase was pre-incubated in Mcllvaine buffer at different pH values (pH 5.0–8.0) and Britton-Robinson buffer (pH 7.0–11.0), at 37 °C for 30 min.

The effects of different concentrations of various metal ions (K^+^, Ca^2+^, Co^2+^, Cu^2+^, Mg^2+^, Mn^2+^, Fe^2+^, Zn^2+^, Pb^2+^, Fe^3+^, Ni^2+^, Hg^2+^, and Ag^+^) and chemical modification reagents including EDTA, DTT, tween 80, βME, triton X-100, SDS, and N-bromosuccinimide (NBS) on Aga-BC7050 activity were examined in McIlvaine buffer at 37 °C for 30 min. The actions of 5, 20, and 100 mM glucose, sucrose, fructose, galactose, and xylose were also studied. The residual activity was measured using pNPGal as the substrate and compared to that with control conditions (the enzyme without any added reagents).

### Protease treatment

To investigate resistance to various proteases, Aga-BC7050 was pre-incubated at 37 °C with proteinase K, subtilisin A, α-chymotrypsin, and trypsin at a ratio of 1:10 (w/w) in 100 mM Tris-HCl buffer (pH 7.4) for 30 min. Residual activity was measured in McIlvaine buffer (pH 6.0) at 55 °C using the standard method. Enzyme activity of the control group (sample without protease treatment) was considered 100% [18].

### Substrate specificity and kinetic parameters

To examine the substrate specificity, Aga-BC7050 was used to hydrolyze different synthetic and natural substrates. To ascertain substrate specificity for natural substrates, the amount of released reducing sugar was determined using the method described by Miller with 3, 5-dinitrosalicylic acid [28]. To measure glucose release, a glucose oxidase assay kit (from Beijing Applygen Technologies Inc.) was used. For melibiose, 5 mM substrate was incubated with appropriately diluted enzyme in McIlvaine buffer for 30 min at 55 °C. One unit of α-galactosidase activity was defined as the amount of enzyme that released 1 μmol of glucose per minute under the assay conditions. The α-galactosidase activity with raffinose, stachyose, guar gum, and locust bean gum was determined by measuring the amount of reducing sugars released, as above. One unit of enzyme activity was defined as the amount of enzyme releasing 1 μmol of reducing sugar (here galactose) per minute. The reaction was terminated by heating in boiling water for 10 min. Data shown are mean values ± SD from at least three independent assays.

The kinetic parameters (*K*m, *V*max, and *k*cat) of Aga-BC7050 against various substrates were also measured. Measurements were performed with pNPGal (0.5–5 mM), melibiose (1–10 mM), raffinose (3–10 mM), and stachyose (2–8 mM) as substrates in McIlvaine buffer (pH 6.0) at 55 °C. From at least three independent experiments, the data were plotted using Michaelis-Menten method with GraphPad Prism v5.0 software [13, 14].

### Hydrolysis properties

To investigate the enzymatic hydrolysis of natural substrates including melibiose, raffinose, and stachyose, 1.0 U/mL enzyme was incubated with 5 mg/mL of substrate for different periods of time in McIlvaine buffer (pH 6.0) at 55 °C. Aliquots were boiled for 10 min at predetermined time-intervals to terminate the reaction, then the reaction products were analyzed on TLC plates using the Shivam and Mishra method [29]. The reaction products were spotted 1 cm from the bottom of a silica gel 60F 254 plate and was developed using a three-phase solvent system of propanol:acetic acid:water at 1:1.5:0.1 (v/v/v). The silica gel plates were sprayed with a methanol/sulfuric acid mixture at a ratio of 95:5 (v/v), and then the plates were heated in an oven at 135 °C for 15 min to detect the saccharides [13].

## Results and discussion

### Molecular cloning and sequence analysis of Aga-BC7050

To obtain efficient α-galactosidases from *Bacillus* species, we searched the corresponding genomes and selected seven strains from different species harboring the predicted α-galactosidase-encoding gene. We cultivated the seven *Bacillus* strains in nutrient medium for 12 h and then collected and disrupted the cells by sonication to obtain the crude enzyme. The crude enzymes were investigated for their α-galactosidase activity at background expression levels and tested the effect of trypsin treatment on enzyme activity. As a result, the α-galactosidases from *B*. *circulans* and *B*. *korlensis* were slightly resistant to trypsin, but only that of *B*. *coagulans* possessed strong resistance (summarized in S1 Table). *B*. *coagulans* is a probiotic used in dietary supplements and food that confers health benefits to the host [30]. By analyzing the published genomes of *B*. *coagulans* in the GenBank database, we found some predicted α-galactosidase genes suggesting that this species probably can express a functional α-galactosidase. To our knowledge, there is no report of the characterization or cloning of an α-galactosidase-encoding gene from *B*. *coagulans*. Such research could help us to further understand the mechanism associated with probiotics. Due to the low background expression of α-galactosidase in *B*. *coagulans* ATCC 7050, it was not convenient for us to further study its properties using its natural host, and thus we cloned and overexpressed the associated gene in *E*. *coli*.

**Fig 1:**
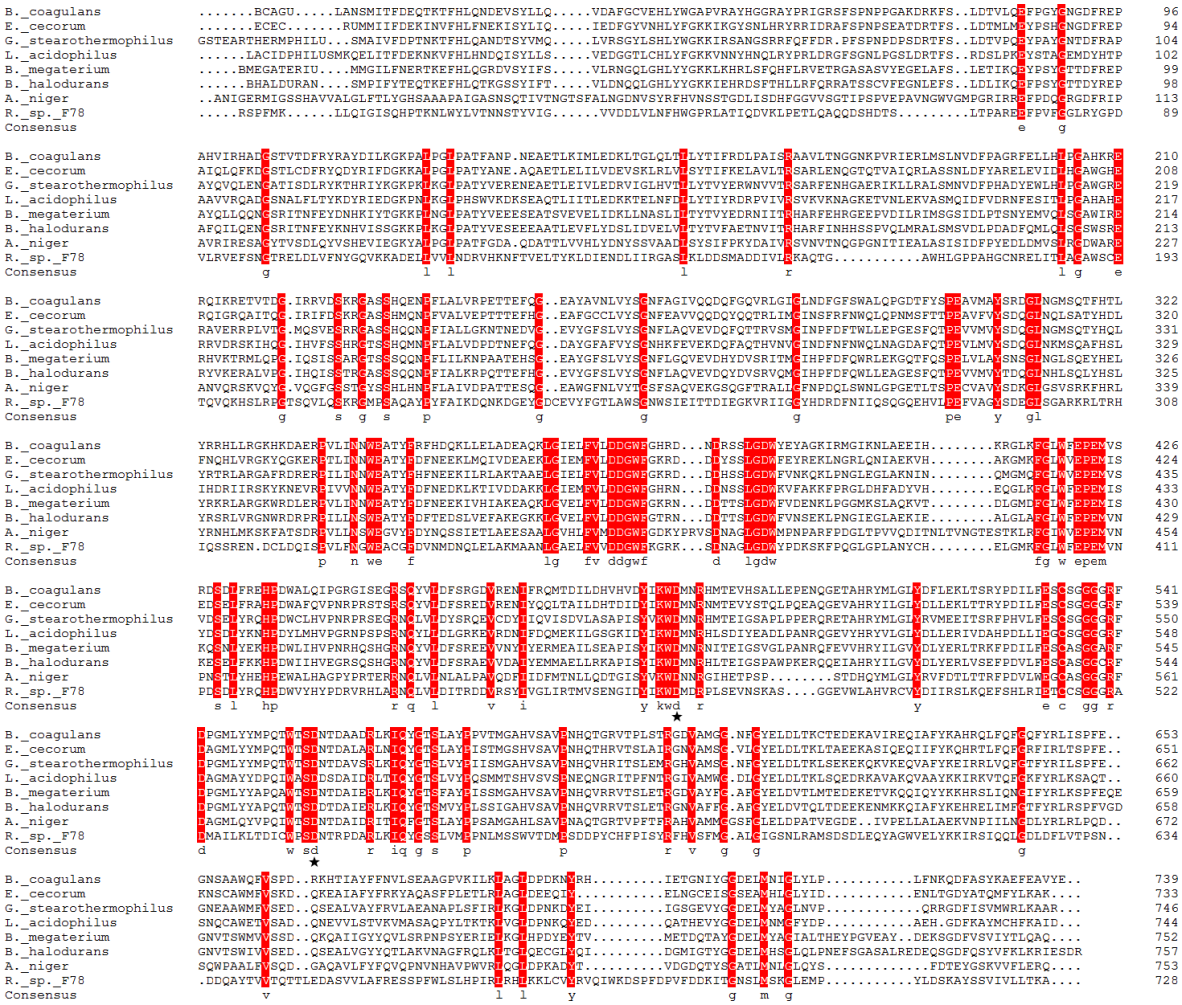
Amino acid sequence alignment of Aga-BC7050 with other glycoside hydrolase (GH) family 36 α-galactosidases. The sequences are indicated with their genus initials, including *Enterococcus cecorum*, *Geobacillus stearothermophilus*, *Lactobacillus acidophilus*, *Bacillus megaterium*, *Bacillus halodurans*, *Aspergillus niger*, and *Rhizopus* sp. The gaps are indicated by hyphens. Similar or identical amino acid residues at the same position are highlighted. The asterisks show putative catalytic residues.

The complete DNA sequence of Aga-BC7050 gene is 2193 bp in length (GenBank, gi: 753712783). The putative protein fused with a His-tag was 753 amino acids with a theoretical molecular weight of 85.62 kDa and a pI of 6.68. α-Galactosidases are highly conserved in *B*. *coagulans* (S2 Table). According to multiple protein sequence alignments, Aga-BC7050 had higher homology with bacterial GH family 36 α-galactosidases from *Enterococcus cecorum* (Accession No. WP_047338472.1; identity 61%), *Geobacillus stearothermophilus* (Accession No. AAD23585.1; identity 57%), *Lactobacillus acidophilus* (Accession No. WP_013642253.1; identity 51%), *Bacillus megaterium* (Accession No. WP_060745422.1; identity 51%), and *Bacillus halodurans* (Accession No. WP_010898379.1; identity 50%) than with fungal α-galactosidases from *Aspergillus niger* (Accession No. Q9UUZ4.1; identity 41%) and *Rhizopus* sp. (Accession No. ACM48349.1; 37%) (Fig 1) and had even lower homology (less than 10%) with α-galactosidases from the GH family 27. Sequencing and conceptual translation showed that Aga-BC7050 had high sequence similarity to other reported α-galactosidases of GH family 36. Aga-BC7050 also had identities of < 41% compared to the reported protease-resistant α-galactosidases RmGal36 (33%) [13], Aga-F75 (41%) [12, 31], Aga-MJ11 (34%) [17], Aga-F78 (37%) [11, 32], Aga-S27 (33%) [19], and AgaAJB13 (34%) [18]. This indicates that Aga-BC7050 represents a new protease-resistant α-galactosidase from *B*. *coagulans* belonging to the GH family 36. The homology-modeled structure of Aga-BC7050 was predicted to have a catalytic domain with a (β/α)_8_-barrel fold, in which the predicted active sites in Aga-BC7050 were the nucleophile Asp477 and acid/base Asp547 (Fig 1).

### Protein expression and purification of Aga-BC7050

**Fig 2:**
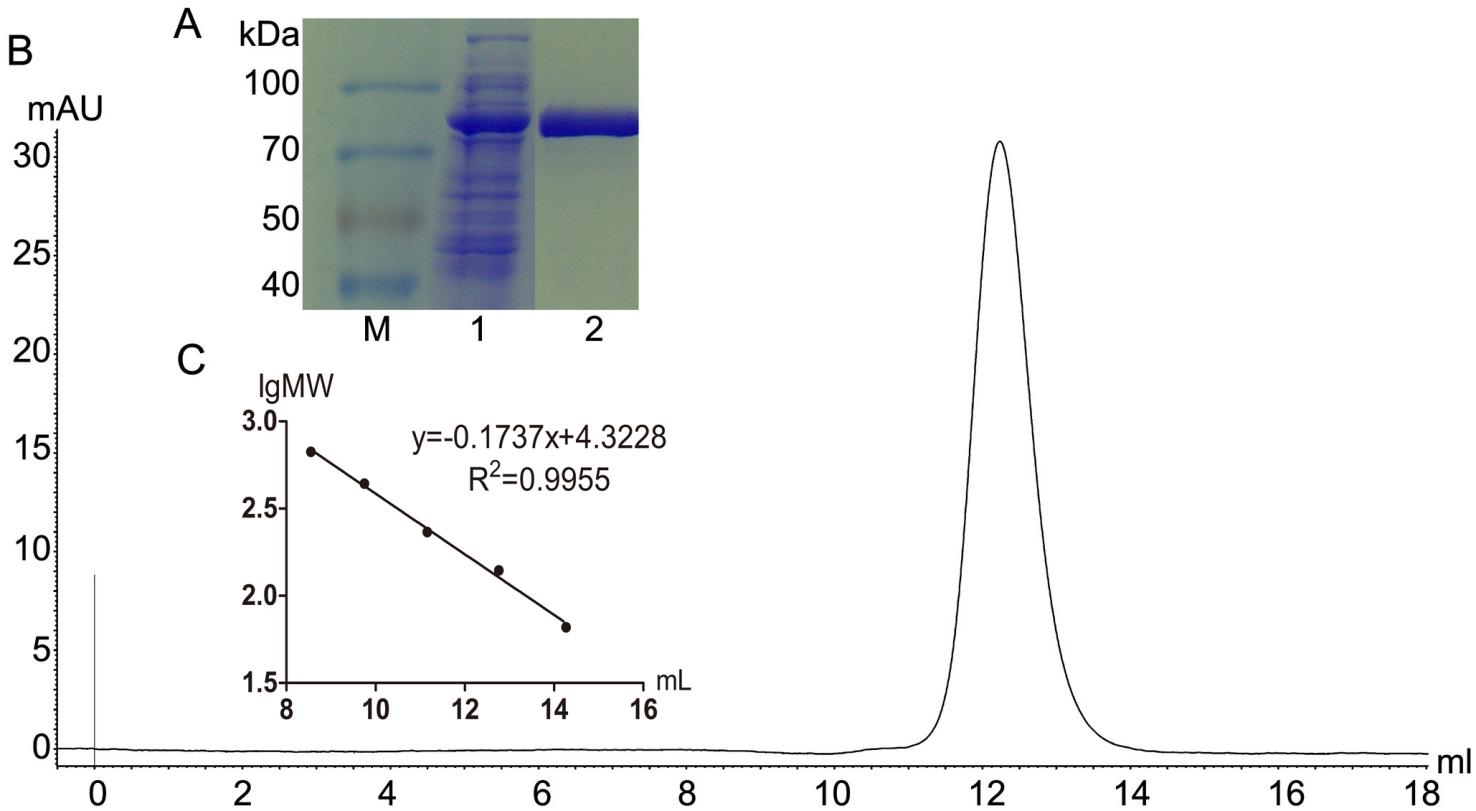
Determination of the molecular weight and oligomeric state of Aga-BC7050 by analytical gel filtration. (A) SDS-PAGE analysis of Aga-BC7050. Lanes: M, protein molecular markers; 1, crude lysate; 2, purified Aga-BC7050. (B) Elution profile from the analysis of purified Aga-BC7050. (C) Calibration curve using protein standards: albumin, 66.0 kDa; lactate dehydrogenase, 140.0 kDa; catalase, 232.0 kDa; ferritin, 440.0 kDa; thyroglobulin, 669.0 kDa.

In the host cell *E*. *coli* BL21, the gene encoding Aga-BC7050 was successfully expressed under the control of the T7 promoter and *lac* operator. In liquid culture, Aga-BC7050 was produced as a soluble intracellular enzyme upon IPTG induction. The product was released into the supernatant after disruption by sonication and centrifugation of the cells. The resulting His-tag fusion of Aga-BC7050 was purified by Ni^2+^-NTA metal chelating affinity chromatography. The activity of Aga-BC7050 was 80.55 U/mg with pNPGal. By SDS-PAGE, only one band was detected, and the protein was estimated to be approximately 85 kDa under reducing conditions, consistent with its predicted molecular weight (Fig 2A). By gel filtration chromatography, the native molecular weight was determined to be 168 kDa (Fig 2B and 2C), indicating that Aga-BC7050 exists as a homodimer in solution, similar to bacterial and fungal α-galactosidases of GH family 36 [33, 34]. Other GH family 36 α-galactosidases were found to be octamers [1, 35], tetramers [36, 37], and trimers [20, 38], whereas plant alkaline α-galactosidases [39], and α-galactosidase from the bacterium *Thermotoga maritima* [40] were found to be monomers. To date, α-galactosidases from *Bacillus* have only been found to be tetramers (*B*. *stearothermophilus*) [36] and trimers (*B*. *megaterium*) [20], indicating that Aga-BC7050 might have different properties and mechanisms.

### Biochemical properties of Aga-BC7050

**Fig 3:**
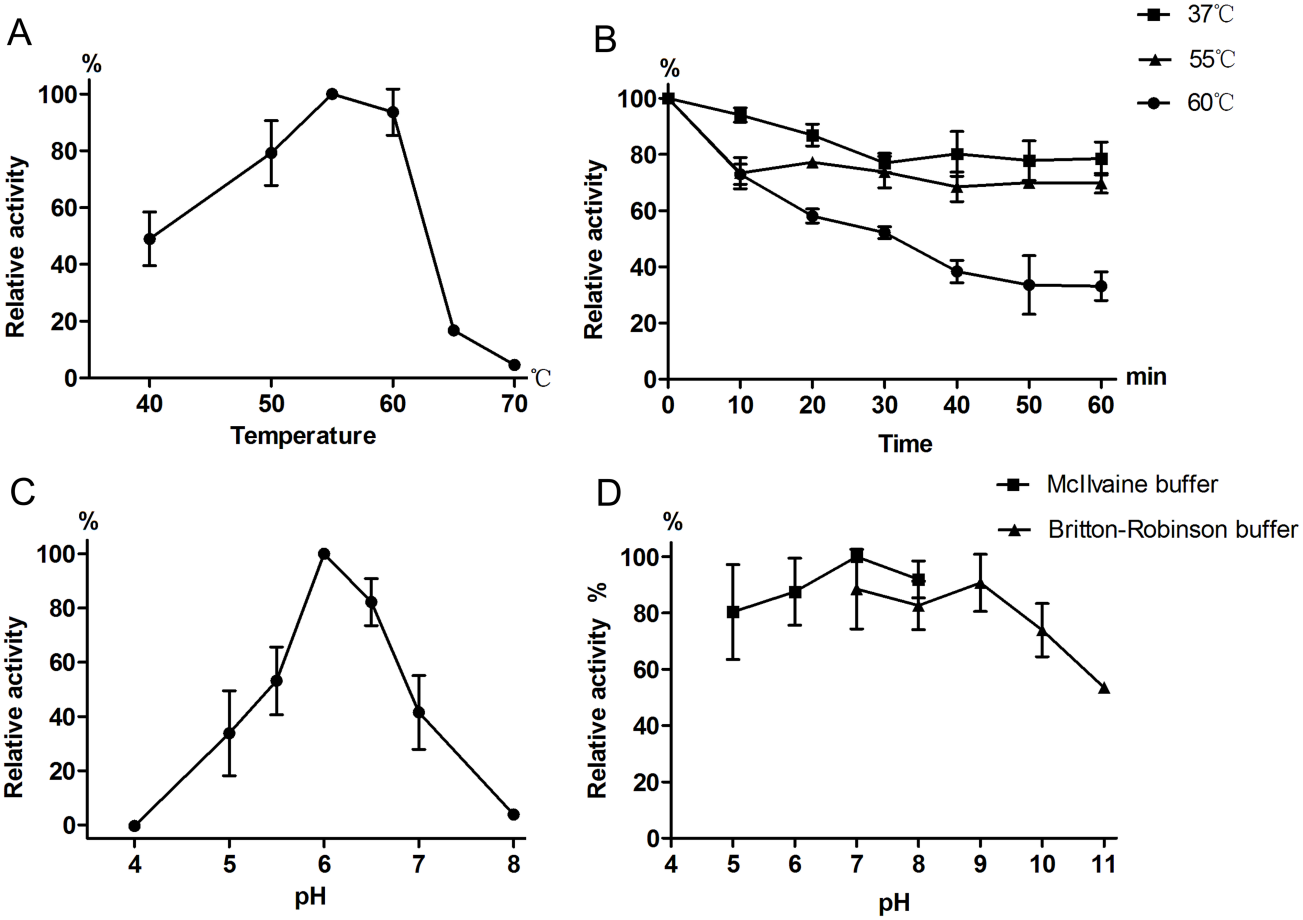
Effect of temperature and pH on the enzymatic properties of purified Aga-BC7050. (A) Optimal temperature, (B) thermostability assay, (C) optimal pH, (D) pH stability assay. The effect of temperature was determined using different temperatures and McIlvaine buffer (pH 6.0). The pH profiles were measured at 55 °C with different buffers including McIlvaine (pH 4.0–8.0) and Britton-Robinson (pH 7.0–11.0). Error bars represent standard deviations.

The effect of temperature on Aga-BC7050 activity is presented in Fig 3A and 3B; α-galactosidase activity increased up to 55 °C (the optimum temperature) and then decreased suddenly above 60 °C. According to thermostability assays, Aga-BC7050 retained approximately 50% of residual activity after incubation in McIlvaine buffer (pH 6.0) at 60 °C for 30 min. The enzyme was most active at 55 °C and had a good thermal stability (half-life > 30 min at 60 °C). A similar result was also reported by Patil for α-galactosidase from *B*. *megaterium* [41]. The thermostability of α-galactosidase has an added advantage for food processing, with respect to minimizing contamination caused by mesophilic organisms.

The optimum pH for the enzymatic hydrolysis of pNPGal was 6.0 in McIlvaine buffer (Fig 3C). Moreover, Aga-BC7050 was highly stable at the pH range of 5.0–10.0, retaining more than 70% of its catalytic activity after 30 min of pre-incubation (Fig 3D). The optimum pH for the enzyme was 6.0 and the pH stability range was 5.0–10.0. In general, the optimal pH for α-galactosidases from bacteria are often slightly alkaline (pH 6.0–7.5) [26, 41], but it is acidic (pH 3–5) for the α-galactosidases from fungi [13, 27, 42]. Since the pH of soymilk is approximately 6.2–6.4, an acidic α-galactosidase is not suitable for hydrolysis [43], and lowering the pH of soymilk causes soy proteins to precipitate, leaving a sour taste [41]. Thus, since Aga-BC7050 has an optimum pH of 6.0, it is appropriate for treating the RFOs present in soymilk.

### Effects of metal ions and specific chemical reagents on Aga-BC7050

The effects of various metal ions and chemical reagents on purified Aga-BC7050 were tested (Table 1). Ag^+^ and Hg^2+^ completely inhibited enzyme activity, whereas the other metal ions had no evident effect on Aga-BC7050, including Cu^2+^ and Fe^3+^. In contrast, many other α-galactosidases are partially or entirely inhibited by Cu^2+^ and Fe^3+^ [9, 16, 41]. No loss in α-galactosidase activity was observed when chemical reagents were present, except for SDS, which completely inhibited activity. This could be because SDS is a strong denaturing agent, and could destroy the tertiary structures of proteins [15]. No obvious loss in activity was observed when the metal chelating agent EDTA was present, indicating that Aga-BC7050 is not a metal-based enzyme [16]. Aga-BC7050 was completely inactivated at an NBS concentration from 0.1 to 10 mM, which indicates a tryptophan residue is located in the active site and is indispensable for Aga-BC7050 enzymatic activity [9]. This is reminiscent of the finding that α-galactosidase from a strain of *B*. *stearothermophilus* is also completely inactivated by NBS [44].

**Table 1:** Effects of different metal ions and specific chemical reagents on the activity of Aga-BC7050.

Substance	Relative activity (%)			
	10 mM	5 mM	2.5 mM	1.25 mM
**K**^**+**^	108.28 ± 0.14	98.88 ± 0.14	100.16 ± 0.23	107.54 ± 0.14
**Ca**^**2+**^	127.39 ± 0.20	106.86 ± 0.27	117.93 ± 0.23	100.92 ± 0.12
**Co**^**2+**^	109.27 ± 0.17	110.67 ± 0.22	119.17 ± 0.14	120.27 ± 0.06
**Cu**^**2+**^	91.05 ± 0.18	101.80 ± 0.24	97.26 ± 0.14	99.73 ± 0.18
**Mg**^**2+**^	94.87 ± 0.04	96.55 ± 0.02	99.0 ± 0.10	100.20 ± 0.06
**Mn**^**2+**^	98.44 ± 0.08	98.80 ± 0.09	98.11 ± 0.05	98.66 ± 0.10
**Fe**^**2+**^	94.23 ± 0.09	90.83 ± 0.09	93.44 ± 0.07	88.94 ± 0.07
**Zn**^**2+**^	108.18 ± 0.18	116.51 ± 0.13	100.18 ± 0.03	109.41 ± 0.16
**Pb**^**2+**^	119.38 ± 0.06	113.25 ± 0.06	111.09 ± 0.05	113.81 ± 0.04
**Fe**^**3+**^	102.53 ± 0.05	113.26 ± 0.06	116.59 ± 0.03	107.66 ± 0.05
**Ni**^**2+**^	114.36 ± 0.04	110.46 ± 0.04	104.46 ± 0.12	99.36 ± 0.04
**Hg**^**2+**^	0.00	0.00	0.00	0.00
**Ag**^**+**^	0.00	0.00	0.00	0.00
**EDTA**	145.96 ± 0.24	127.82 ± 0.17	122.50 ± 0.12	133.14 ± 0.31
**DTT**	101.82 ± 0.04	93.47 ± 0.06	98.54 ± 0.04	107.00 ± 0.06
**Tween 80**	134.01 ± 0.08	134.65 ± 0.09	136.04 ± 0.07	135.71 ± 0.10
**βME**	115.00 ± 0.10	109.82 ± 0.17	108.94 ± 0.13	108.53 ± 0.18
**Triton X-100**	144.54 ± 0.11	145.80 ± 0.13	148.74 ± 0.15	149.50 ± 0.15
**SDS**	0.00	0.00	0.00	0.00

The control as 100% was the α-galactosidase activity without incubation with metal ions or chemical reagents. Data shown are the means ± SD (n = 3).

The effect of various low molecular sugars on Aga-BC7050 was also assessed, and results are shown in Table 2. When concentrations increased from 5 to 100 mM, the activity of Aga-BC7050 was not affected by glucose, sucrose, fructose, or xylose. Likewise, galactose did not distinctly effect the activity at a concentration of 5–20 mM. Nevertheless, as the galactose concentration increased to 100 mM, Aga-BC7050 activity decreased by 24.4%. In contrast, *B*. *megaterium* α-galactosidase was appreciably influenced by xylose, glucose, and galactose at concentrations < 100 mM [20]. In addition, the commercial α-galactosidase from green coffee bean was partially inhibited by fructose, galactose, and xylose, and especially galactose (reduced by 89%) (S1 Fig). d-Galactose, one of the reaction products on hydrolysis of α-d-galactosides by α-galactosidase, is a potent inhibitor of microbial α-galactosidases [45, 46], except α-galactosidase from *Streptomyces griseoloalbus* [47]. During soy processing and in the beet sugar industries, α-galactosidases are generally largely inhibited by their hydrolysis products (such as galactose and sucrose) [13, 48]. The exceptional tolerance to metal ions and high sugar concentrations suggests that Aga-BC7050 will be more beneficial for these applications.

**Table 2:** Effects of sugars on the activity of Aga-BC7050.

Saccharide	Relative activity (%)		
	5 mM	20 mM	100 mM
**Glucose**	100.6 ± 4.7	100.5 ± 3.4	102.2 ± 6.8
**Sucrose**	98.6 ± 1.2	99.3 ± 2.8	101.8 ± 3.6
**Fructose**	104.8 ± 6.7	113.1 ± 12.7	107.6 ± 3.9
**Galactose**	98.7 ± 10.4	96.1 ± 7.5	75.6 ± 3.8
**Xylose**	99.3 ± 4.7	110.6 ± 13.9	101.4 ± 3.4

The data represent the percent mean ± SD (n = 3).

### Resistance to proteases

**Fig 4:**
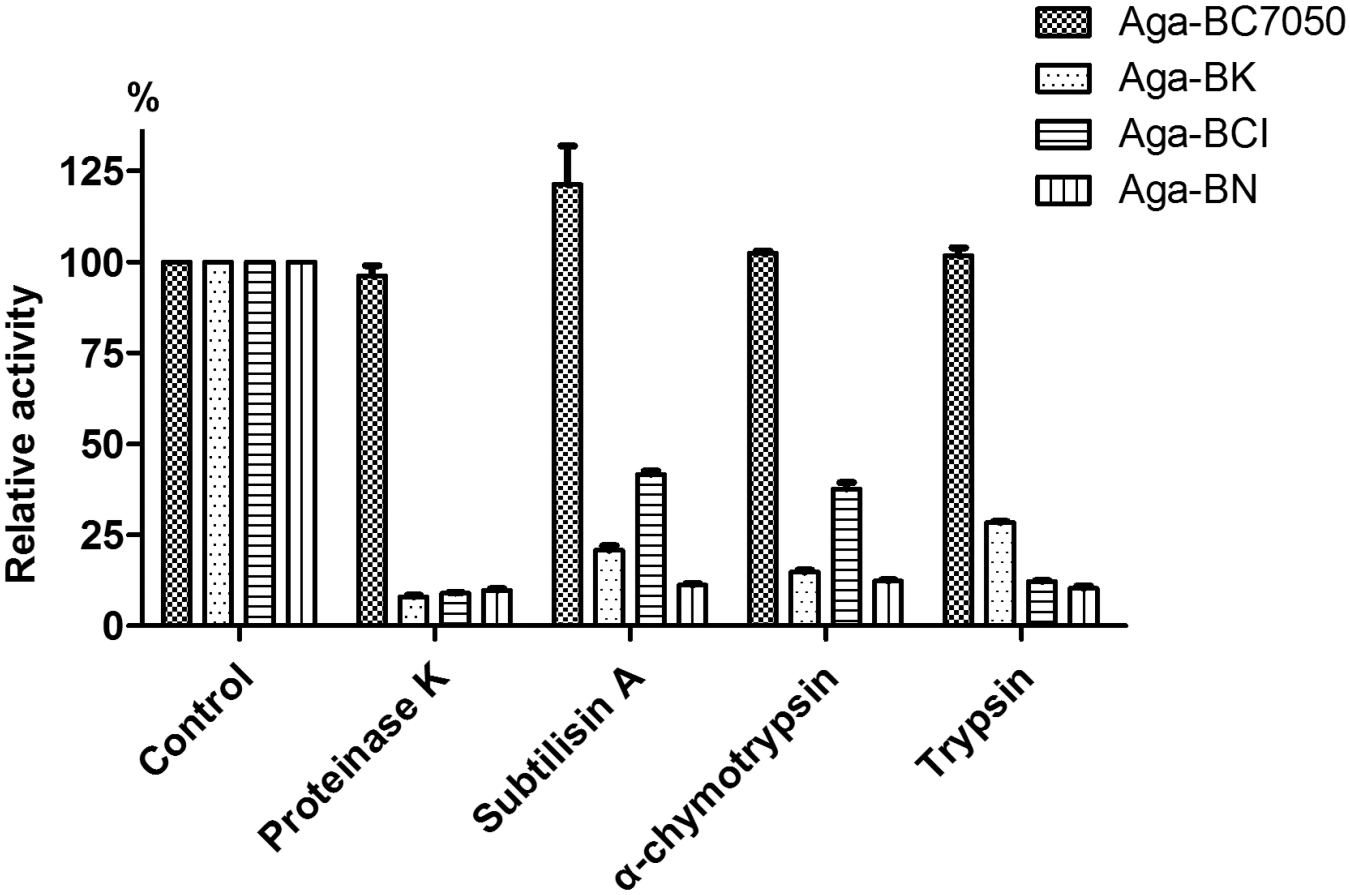
Effect of proteases on the enzyme activity of Aga-BC7050, Aga-BK, Aga-BCI, and Aga-BN. The purified enzymes were pre-incubated with protease at a 1:10 (w/w) ratio in 100 mM Tris-HCl buffer (pH 7.4) at 37 °C for 30 min, and the enzyme activity was then measured under standard conditions. The activity of α-galactosidase without protease was set to 100%. Values represent the mean ± SD (n = 3).

To investigate the resistance to various proteases, the α-galactosidase Aga-BC7050 was treated with four different proteases (proteinase K, subtilisin A, α-chymotrypsin, and trypsin). In order to ensure that the experiment was scientifically valid and accurate, we attempted to overexpress the other six α-galactosidases from *Bacillus* in *E*. *coli* as controls. Three of the six were expressed successfully and we compared their protease resistance with that of Aga-BC7050. The results showed that Aga-BC7050 possessed excellent protease resistance after incubation with several proteases. After treatment with proteinase K at 37 °C for 0.5 h, it retained 96.2% of its initial activity; however, Aga-BK (*B*. *korlensis*), Aga-BCI (*B*. *circulans*) and Aga-BN (*B*. *niacini*) retained only 8.0, 9.0, and 9.7% of activity, respectively (Fig 4). With subtilisin A treatment, the activity of Aga-BC7050 increased marginally (121.2%). After this treatment, Aga-BCI retained 41.6% of its activity, whereas Aga-BK and Aga-BN retained only 20.8% and 11.2%, respectively. After hydrolysis for 0.5 h using α-chymotrypsin, Aga-BC7050 was virtually unaffected (102.4%); however, Aga-BCI activity decreased to 37.6%, which was still higher than that of Aga-BK (14.8%) and Aga-BN (12.3%). Similarly, with trypsin, Aga-BC7050 was unaffected (101.8%), whereas Aga-BK, Aga-BCI, and Aga-BN retained only 28.3, 12.2, and 10.3% of activity, respectively.

According to other reports, bacterial α-galactosidases with protease resistance were also isolated from *Pedobacter nyackensis* MJ11 [17], a new *Sphingomonas* strain [18], *Streptomyces* sp. S27 [19], and *B*. *megaterium* [20]. Compared to these bacterial α-galactosidases, Aga-BC7050 displayed enhanced resistance to proteases. The *B*. *megaterium* AgaB exhibited strong resistance to subtilisin A, trypsin, and collagenase type IV, but not α-chymotrypsin and proteinase K (residual activity of 57.5% and 2%, respectively) [20]. The α-galactosidase of the *Sphingomonas* strain was only resistant to trypsin and proteinase K [18]. That of *Pedobacter nyackensis* MJ11 retained less than 30% of its activity in the presence of trypsin, and proteinase K inhibited greater than 80% of the activity of α-galactosidase from *Streptomyces* sp. S27 [17, 19]. To remove anti-nutritional factors and improve nutritional value and digestibility, α-galactosidases are often combined with proteases as additives to make high protein materials more edible in the food and feed industries [11, 17, 49]. After hydrolysis by proteinase K, subtilisin A, α-chymotrypsin, and trypsin, activity staining on native-PAGE indicated no significant structural and size changes for Aga-BC7050 (S2 Fig). The small changes in band position were probably caused by the minor hydrolysis of peripheral amino acid residues; accordingly, we could confirm that its main structure and active center still existed. Due to its remarkable protease resistance, Aga-BC7050 could be a novel candidate additive for food and feed.

### Substrate specificity and kinetic parameters of Aga-BC7050

To investigate the substrate specificity of Aga-BC7050, synthetic substrates pNPGal and natural substrates (melibiose, raffinose, stachyose, guar gum, and locust bean gum) were examined (Table 3). Purified Aga-BC7050 was highly active toward pNPGal, melibiose, raffinose, and stachyose, but had no effect on guar gum and locust bean gum. It was suggested that the huge structure of the polymeric substrates probably made them inaccessible to the active centers of Aga-BC7050 [5]. The substrates melibiose, raffinose, and stachyose had *K*m values of 19.5, 53.7, and 178.5 mM, respectively (listed in Table 4). The lowest *K*m value (1.1) was observed for the substrate pNPGal, indicating that Aga-BC7050 exhibits higher affinity for this compound than for melibiose, raffinose, or stachyose. With respect to the catalytic efficiency (*k*cat/*K*m ratio), the enzyme displayed highest catalytic efficiency with pNPGal, followed by the following compounds in order: melibiose > raffinose > stachyose. This was in accordance with their hydrolysis rates, as determined by TLC (described subsequently).

**Table 3:** Hydrolysis of different substrates by Aga-BC7050.

Substrate	Relative activity (%)
**pNPGal**	100
**Melibiose**	264 ± 0.3
**Raffinose**	28 ± 0.1
**Stachyose**	17 ± 1.5
**Guar gum**	−
**Locust bean gum**	−

Relative activities were calculated compared to pNPGal activity, which was considered 100%.–, no activity was detected.

**Table 4:** Kinetic parameters of Aga-BC7050.

Substrate	*K*m (mM)	*V*max (μmol/min/mg)	*k*cat (s^-1^)	*k*cat/*K*m (s^-1^mM^-1^)
**pNPGal**	1.1 ± 0.2	655.1 ± 55.2	942.2 ± 73.9	844.5
**Melibiose**	19.5 ± 5.6	945.1 ± 26.2	1338.9 ± 37.1	68.8
**Raffinose**	53.7 ± 0.9	1139.6 ± 37.7	1614.4 ± 53.4	30.5
**Stachyose**	178.5 ± 6.5	971.3 ± 45.4	1376.0 ± 64.3	7.7

### Hydrolysis of oligosaccharides by Aga-BC7050

**Fig 5:**
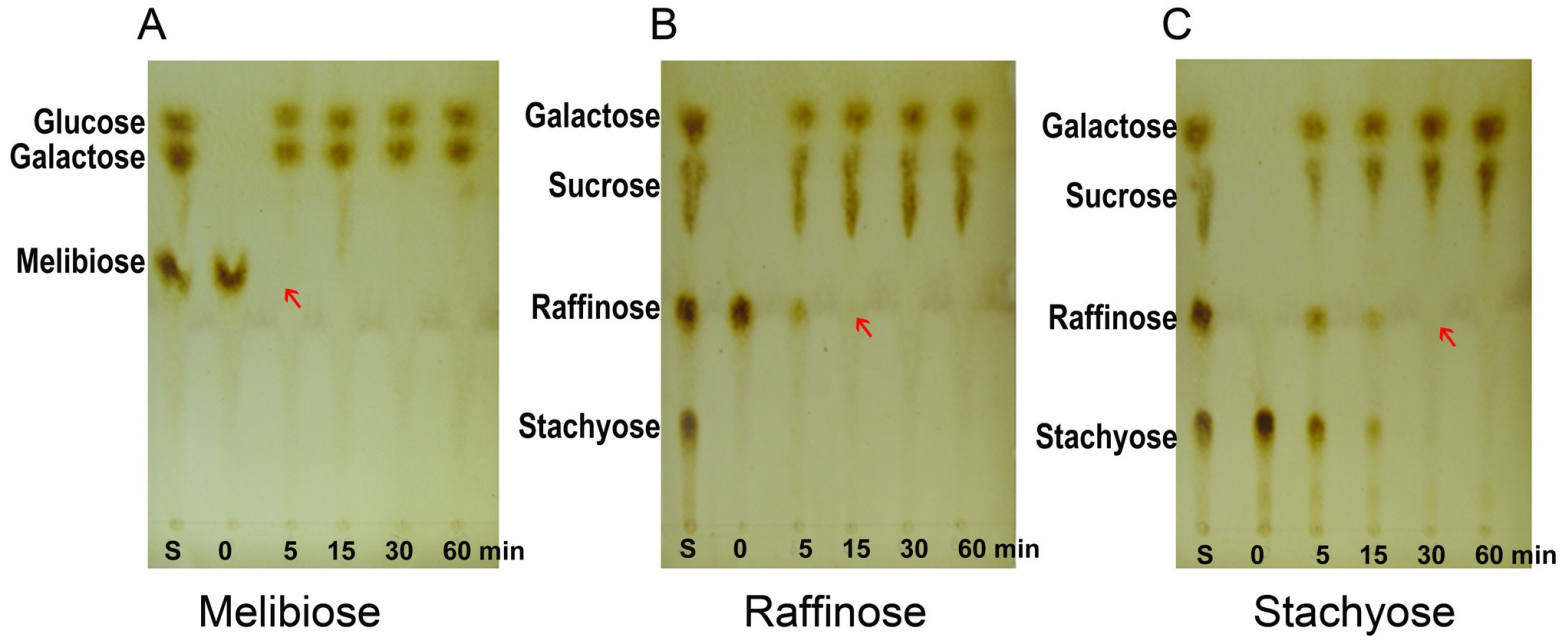
Thin-layer chromatography (TLC) analysis of oligosaccharide degradation by Aga-BC7050. Lane S shows the oligosaccharide standards. Control group (0 min) represents the substrate (melibiose, raffinose, stachyose) without the addition of α-galactosidase.

The hydrolysis of oligosaccharides was detected, and products were analyzed on TLC plates (Fig 5). Melibiose was degraded to glucose and galactose in only 5 min (Fig 5A). Aga-BC7050 effectively hydrolyzed raffinose to sucrose and galactose within 15 min (Fig 5B). During stachyose hydrolysis, stachyose and concurrently generated raffinose were completely hydrolyzed by Aga-BC7050 in only 30 min (Fig 5C). These results indicated that Aga-BC7050 is extremely efficient with respect to the hydrolysis of melibiose, raffinose, and stachyose.

According to previous reports on α-galactosidases from *Bacillus*, α-galactosidase from *B*. *stearothermophilus* rapidly hydrolyzed melibiose in 30 min, but the time required for raffinose and stachyose hydrolysis was relatively long [50]. *B*. *megaterium* AgaB was found to completely hydrolyze raffinose in only 30 min, whereas after 3 h, only 52% of stachyose was hydrolyzed [20]. Based on another report, α-galactosidase from *B*. *megaterium* VHM1 completely hydrolyzed raffinose and stachyose after 1.5 h of incubation at pH 7 and 55 °C [41]. Regarding plant α-galactosidases, that of white chickpea (*Cicer arietinum*) took more than 2 h to hydrolyze raffinose and stachyose [48]. Katrolia *et al*. reported that the fungal α-galactosidase from *Rhizomucor miehei* could complete the effective hydrolysis of raffinose in 15 min and stachyose in 30 min, similar to that observed for Aga-BC7050; however, it did not act efficiently on melibiose [13]. These results indicate that in terms of the removal of non-digestible oligosaccharides, Aga-BC7050 has significant potential in the food industry and in animal feed processing, and especially for beet sugar.

## Conclusion

This paper is the first report of the cloning and characterization of a GH family 36 α-galactosidase from a *B*. *coagulans* strain. The enzyme was cloned and effectively expressed in *E*. *coli*, and its native structure was a homodimer. Aga-BC7050 displayed good thermostability and pH stability. It was also revealed to possess excellent protease resistance and tolerance to low molecular weight sugars. Aga-BC7050 exhibited remarkable hydrolysis of melibiose, raffinose, and stachyose. These excellent properties suggest great potential for Aga-BC7050 in the food, feed, and beet sugar industries for eliminating non-digestible oligosaccharides from beans and beet.

## Supporting information

S1 Fig Effect of sugars on the activity of Aga-BC7050 and Aga-GCB. Aga-GCB is a commercial α-galactosidase from green coffee bean. The concentration of sugars was 100 mM. Data represents the mean ± SD (n = 3).
(TIF)

S2 Fig Aga-BC7050 activity staining on native-PAGE after hydrolysis by proteinase K, subtilisin A, α-chymotrypsin, and trypsin. Aga-BC7050 was pre-incubated with protease at a ratio of 1:10 (w/w) in 100 mM Tris-HCl buffer (pH 7.4) at 37 °C for 30 min. The reaction products were loaded into native-PAGE gels for electrophoresis and then stained with 6-bromo-2-naphthalenyl and Fast Blue B. 1, control (without protease); 2, pre-incubated with proteinase K; 3, pre-incubated with subtilisin A; 4, pre-incubated with α-chymotrypsin; 5, pre-incubated with trypsin.
(TIF)

S1 Table Investigation of the activity and protease resistance of α-galactosidases from *Bacillus*. a: +, α-galactosidase activity;–, no α-galactosidase activity. b: the percentage of remaining enzyme activity after treatment with trypsin,–, 0–10%; +, 10–30%, ++, 30–60%; +++, 60–100%; ND, not detected.
(DOCX)

S2 Table Homology analysis of α-galactosidases from *Bacillus coagulans*. (DOCX)
