# Supplementary figures and images for: A novel α-galactosidase from the thermophilic probiotic Bacillus coagulans with remarkable protease-resistance and high hydrolytic activity
Source: PLoS One. 2018 May 8;13(5):e0197067. doi: 10.1371/journal.pone.0197067 (PMC5940202; doi:10.1371/journal.pone.0197067)

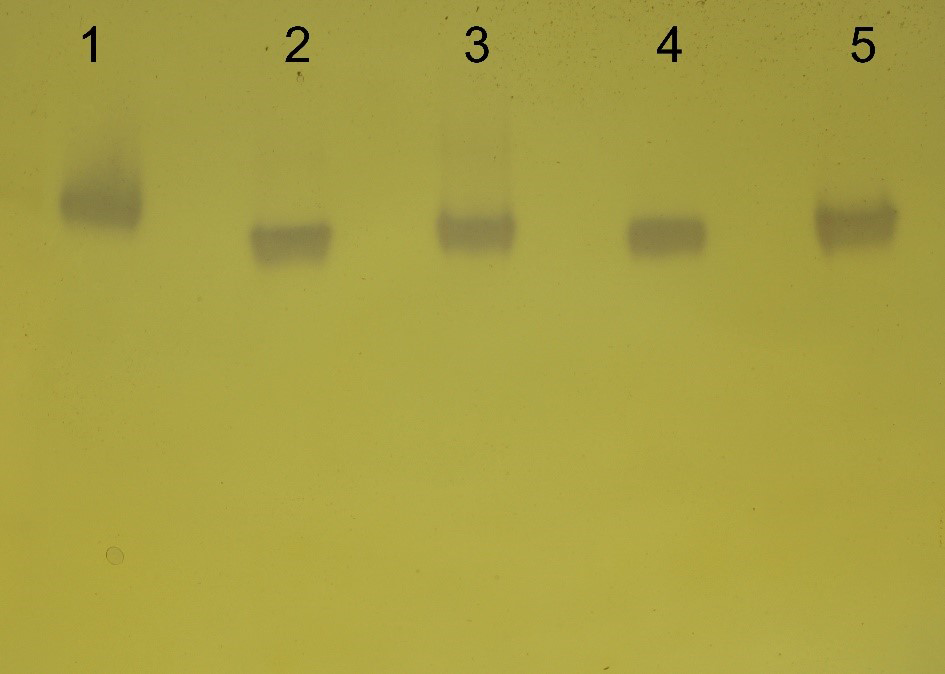

Supplement: S2 Fig — Aga-BC7050 was pre-incubated with protease at a ratio of 1:10 (w/w) in 100 mM Tris-HCl buffer (pH 7.4) at 37 °C for 30 min. The reaction products were loaded into native-PAGE gels for electrophoresis and then stained with 6-bromo-2-naphthalenyl and Fast Blue B. 1, control (without protease); 2, pre-incubated with proteinase K; 3, pre-incubated with subtilisin A; 4, pre-incubated with α-chymotrypsin; 5, pre-incubated with trypsin. (TIF) [file pone.0197067.s002.tif]
