# Supplementary material for: A novel α-galactosidase from the thermophilic probiotic Bacillus coagulans with remarkable protease-resistance and high hydrolytic activity
Source: PLoS One. 2018 May 8;13(5):e0197067. doi: 10.1371/journal.pone.0197067 (PMC5940202; doi:10.1371/journal.pone.0197067)
Supplement: S1 Table — a: +, α-galactosidase activity;–, no α-galactosidase activity. b: the percentage of remaining enzyme activity after treatment with trypsin,–, 0–10%; +, 10–30%, ++, 30–60%; +++, 60–100%; ND, not detected. (DOCX) [file pone.0197067.s003.docx]

| Species | α-galactosidase activity*^a^* | Trypsin resistance*^b^* |
| --- | --- | --- |
| *Bacillus coagulans* ATCC 7050 | + | +++ |
| *Bacillus circulans* CCTCC AB 94026 | + | + |
| *Bacillus niacin* CCTCC AB 95011 | + | – |
| *Bacillus korlensis* CCTCC AB 207172 | + | + |
| *Bacillus kribbensis* CCTCC AB 208184 | + | – |
| *Bacillus muralis* CCTCC AB 209037 | – | ND |
| *Bacillus megaterium* CCTCC AB 92075 | + | – |
