# Supplementary material for: A novel α-galactosidase from the thermophilic probiotic Bacillus coagulans with remarkable protease-resistance and high hydrolytic activity
Source: PLoS One. 2018 May 8;13(5):e0197067. doi: 10.1371/journal.pone.0197067 (PMC5940202; doi:10.1371/journal.pone.0197067)
Supplement: S2 Table — (DOCX) [file pone.0197067.s004.docx]

| Accession | Strains | | Amino acid number | Identity |
| --- | --- | --- | --- | --- |
| WP_026684657.1 | | *Bacillus coagulans* ATCC 7050, CSIL1, GED7749B | 730 | 100% |
| WP_061575419.1 | | *Bacillus coagulans* B4099 | 730 | 99% |
| WP_017553164.1 | | *Bacillus coagulans* H-1 | 730 | 99% |
| WP_061566856.1 | | *Bacillus coagulans* B4098 | 730 | 99% |
| WP_013858558.1 | | *Bacillus coagulans* 2-6 | 730 | 99% |
| WP_019720343.1 | | *Bacillus coagulans* XZL4 | 730 | 99% |
| WP_061565492.1 | | *Bacillus coagulans* B4100 | 728 | 98% |
| WP_061577395.1 | | *Bacillus coagulans* B4096 | 730 | 96% |
| WP_014095945.1 | | *Bacillus coagulans* 36D1 | 730 | 96% |
| WP_046720992.1 | | *Bacillus coagulans* NL01 | 728 | 96% |
| WP_035191081.1 | | *Bacillus coagulans* P38 | 730 | 95% |
| WP_035182298.1 | | *Bacillus coagulans* HM-08, S-lac, LBSC, GBI-30, 6086, Unique IS-2 | 730 | 95% |
| WP_017551588.1 | | *Bacillus coagulans* XZL9 | 730 | 95% |
| WP_071451746.1 | | *Bacillus coagulans* BC-HY1 | 730 | 95% |
